# A two-decade bibliometric analysis (2004–2024) of parental factors in the context of internet gaming disorder research

**DOI:** 10.3389/fpsyt.2026.1815429

**Published:** 2026-05-07

**Authors:** Valentina Novak, Zoran Zoričić, Vladimir Ruf, Sanja Brkić, Marijana Neuberg, Tomislav Meštrović, Krunoslav Capak, Josip Šimić

**Affiliations:** 1 Department of Nursing, University centre Varaždin, University North, Varaždin, Croatia; 2 Faculty of Dental Medicine, University of Zagreb, Zagreb, Croatia; 3 Faculty of Humanities and Social Sciences, University of Mostar, Mostar, Bosnia and Herzegovina; 4 Faculty of Health Studies, University of Mostar, Mostar, Bosnia and Herzegovina; 5 Institute for Health Metrics and Evaluation, University of Washington, Seattle, WA, United States; 6 Croatian Institute of Public Health, Zagreb, Croatia

**Keywords:** bibliometric analysis, autism, bibliometric mapping, family context, internet gaming disorder, parenting

## Abstract

**Objective:**

This is the first targeted bibliometric analysis which explores the development of scientific production on the relationship between parenting and Internet Gaming Disorder (IGD) over twenty years, emphasizing the central role of the family context in the etiology and maintenance of IGD.

**Methods:**

Papers indexed in Scopus and Web of Science databases from 2004 to December 31, 2024, were analyzed using the PRISMA guidelines, the R package Bibliometrix, and VOSviewer. A comprehensive search strategy was developed using Boolean operators to capture variations of parental and gaming-related terminology. Records were exported in BibTeX format and were merged and cleaned to remove duplicates before the analysis. A descriptive bibliometric analysis, bibliometric mapping, and content analysis were conducted to identify trends and thematic clusters. The analysis included 389 publications.

**Results:**

The most cited papers confirm the association of low parental warmth, family dysfunction, and comorbid psychiatric symptoms with a higher risk of IGD. Thematic mapping reveals six dominant clusters covering the conceptualization and diagnosis of IGD, parental mediation and virtual environment, psychological vulnerability and mental health, parenting and attachment, parenting styles and self-control, and problematic screen-related behaviors, and a strong concentration of publications in China, Germany, and the USA. The analysis also revealed an increase in publication output after 2013, with a notable acceleration following the inclusion of gaming disorder in the International Classification of Diseases 11th Revision (ICD-11).

**Conclusion:**

The bibliometric analysis reveals the rapid growth of research on parenting and IGD, highlighting the multifactorial nature of the disorder where dysfunctional family relationships increase risk, while supportive ones reduce it. Despite progress, longitudinal studies are needed for better understanding of causality and interventions.

## 1 Introduction

Internet Gaming Disorder (IGD) has been recognized as a growing public health problem worldwide, contributed to by the rapid development of technologies, increasing availability of gaming, as well as increasingly sophisticated and immersive characteristics of video games. While the majority of video game users engage recreationally without significant negative consequences, a portion of individuals develop patterns of excessive and compulsive gaming associated with significant functional impairments in academic, work, family, and social functioning (1, 2) - which the World Health Organization (WHO) recognized in 2019 and included in the 11^th^ revision of the International Classification of Diseases (ICD-11) under the name of “gaming disorder”. Gaming disorder can be caused by the use of online and offline video games and is defined as a pattern of repeated or persistent gaming characterized by impaired control over gaming, giving increased priority to gaming over other activities, and continuing gaming despite negative consequences, with symptoms lasting at least 12 months (3).

The American Psychiatric Association (APA) recognized these behavioural changes earlier and in 2013 included IGD in the DSM-5 as a condition requiring further empirical research, with very similar but more detailed criteria. The nine APA criteria include preoccupation with gaming, withdrawal symptoms, tolerance, loss of interest in other activities, deception of the environment, continuation of gaming despite problems, loss of significant relationships, escapism as a reason for gaming, and unsuccessful attempts to reduce gaming (4).

The criteria for defining the disorder from umbrella organizations are similar, with WHO describing them more precisely and requiring that for diagnosing IGD, the criteria (at least five out of the nine listed) must be present for at least one year (5).

IGD prevalence has been recorded in various age groups in studies worldwide (6), but it is higher in children and adolescents. There are differences in research results attributed to different diagnostic criteria, measurement instruments used, research methodology, and cultural context, but it is approximately 3-10% (7).

The meta-analysis by Fam et al. from 2018, synthesizing findings across three decades, shows that pooled estimates of IGD prevalence among adolescents are around 4.6%, with substantial variability depending on the diagnostic criteria and measurement instruments used (8). A more recent meta-analysis by Satapathy et al. (2025) focusing on adolescents, which synthesized 84 studies and >640,000 participants, reported a pooled prevalence of 8.6% for IGD and highlighted marked heterogeneity and increasing estimates over time (2) region-specific evidence suggests higher prevalence in certain contexts: a systematic review and meta-analysis of Chinese adolescents estimated IGD prevalence at about 10%, with moderators including sex, sample size, and study year (9). Taken together, these newer findings support the conclusion that IGD epidemiology in young people is heterogeneous, with potentially higher rates in specific subgroups (e.g., gamers in certain genres) and sex-related differences, further justifying a focus on parental and family factors as key sources of risk variability and important targets for prevention and intervention.

The etiology of IGD is complex and involves the interaction of individual, psychological, family, and environmental factors. At the user level, risk factors include elevated impulsivity, aggression, lower self-esteem, emotional dysregulation, and the presence of depressive and anxiety symptoms, as well as gaming habits – longer daily gaming time, greater financial investment in video games, early age of gaming onset, and preference for genres like FPS and MMORPG games further increase the likelihood of developing IGD (10–12).

The family context is recognized as a significant etiological framework, where dysfunctional family functioning, weaker connectedness, conflictual relationships, and lower parental warmth are associated with a higher risk of IGD, while warmer relationships, parental involvement, and supervision reduce the risk (13). Family context is increasingly recognized as an important factor in the etiology, with particular emphasis on the quality of parent-child relationships and parental supervision and setting boundaries related to video game playing. Schneider et al. (13) in a systematic review of family factors in IGD link the level of conflict, emotional closeness, family stress, and dysfunctional communication with the risk of developing IGD, while warmer and supportive relationships are recognized as protective factors, although effects may vary depending on gender, age, and cultural context (13).

To comprehensively understand the development and scientific impact of research on the relationship between parenting and IGD, a comprehensive bibliometric analysis of scientific production on parental and family aspects of IGD (based on data from Scopus and Web of Science databases) is needed. Since bibliometric analyses have proven to be an objective tool for examining research trends, citation networks, geographic distribution of papers, and thematic clusters in the mentioned field, such an analysis helps in identifying key themes, authors and journals, and also provides insight into the development of the field and its interdisciplinary connections between psychology, psychiatry, health sciences, and information/communication studies.

To date, several bibliometric studies have been conducted examining scientific activity in the field of IGD (14–17). Mentioned papers analyzed global trends in the general field of IGD without focusing on the influence of parental factors, where parents, parenting, and related terms appear only marginally as secondary clusters of keywords, if at all. Some bibliometric analyses, such as Cheng et al. (18) or Muflih et al. (19), focused on a broader search scope, i.e., internet addiction disorder, or generally investigated internet use disorders as, e.g., Le et al. (20).

All the mentioned analyses have in common that they retrieved data from only one of the international databases (all except Hanoum et al. (21), which retrieved data from Scopus, retrieved data exclusively from Web of Science), thereby limiting the set of relevant and available studies.

Upon checking the literature and the mentioned bibliometric studies, to the best of our knowledge there are no studies aimed at depicting the relationship between parenting and IGD. This research, unlike previous ones, will include a bibliometric analysis of the literature with a focus on the topic of the relationship between parenting and IGD. Therefore, the aim of this study is to identify hotspots and trends in research on the relationship between parenting and IGD to provide valuable information for future research.

This study, therefore, represents the first attempt at a bibliometric synthesis linking the domain of parenting and IGD, with the aim of mapping research trends, the most influential authors and journals, and identifying thematic areas and gaps in previous scientific production. We have developed the following research questions:

1. What is the volume, scope, and trends in scientific productivity in the field of the relationship between parenting and IGD?
2. How is the literature on the relationship between parenting and IGD distributed among leading sources, countries, institutions, and authors?
3. What are the most prominent research themes on the relationship between parenting and IGD; what is the connectivity among them, and how have they evolved over time?

## 2 Materials and methods

### 2.1 Bibliographic databases and data sources

Data extraction was conducted on 10 October 2025, and the analysis and examination of the received data were performed in October and November. Data from the existing literature were obtained and exported from Scopus (Scopus, Elsevier, Amsterdam, Netherlands) and Web of Science (Clarivate, London, United Kingdom). Notably, our study covers the entire Web of Science database, providing comprehensive and systematic coverage.

To achieve the broadest inclusion of publications dealing with the relationship between parental factors and IGD, Scopus and WoS were selected as primary bibliographic and peer-reviewed data sources. Both databases are highly structured, comprehensive, and compatible with bibliometric tools for extraction, processing, and merging data in multiple formats. Their standardized metadata provide detailed and reliable information essential for quantitative bibliometric analysis (22).

### 2.2 Search strategy and query

First, we developed a comprehensive search string using terms from relevant articles, documents, guidelines, and recommendations. Then, we refined the search through iterative testing, exploring combinations and individual terms to cover most active synonyms related to the relationship between parenting and IGD. A challenge in creating the search string was the appearance of certain acronyms in different contexts (IGD as a type of immunoglobulin and VGA as Virtual Gender Approach; excluding them from the string would omit several important papers that do not use full disease/disorder names), so we used all standard Boolean operators.

The final search queries were constructed using Scopus TITLE-ABS-KEY and WoS TS filters as follows: TITLE-ABS-KEY (“Parent*” AND (“Internet gaming disorder” OR “IGD” OR “video game addiction” OR “VGA” OR “problematic video game use” OR “negative effects of video games” OR “gaming disorder” OR “gaming overuse”) AND NOT immun*) and TS=(“Parent*” AND (“Internet gaming disorder” OR “IGD” OR “video game addiction” OR “VGA” OR “problematic video game use” OR “negative effects of video games” OR “gaming disorder” OR “gaming overuse”) NOT immun*).

In the scientific literature, a range of terms-including IGD, “gaming disorder,” “video game addiction” (VGA), “problematic video game use,” “gaming overuse,” and related expressions such as “negative effects of video games”- are employed to denote the same or at least closely related phenomenon of excessive video gaming that leads to clinically significant distress or impairment in individual functioning. In the DSM-5/DSM-5-TR, this phenomenon is proposed under the name Internet gaming disorder as a condition for further study, emphasizing persistent and recurrent gaming (primarily via the internet) accompanied by significant impairment in personal, family, social, or academic/occupational functioning. In contrast, the ICD-11 introduces the term gaming disorder, yet its descriptive core is nearly identical - the disorder is defined by impaired control over gaming, prioritization of gaming over other activities, and continuation or escalation of gaming despite negative consequences, in the presence of clinically significant dysfunction. Outside classification manuals, authors frequently use terms such as video game addiction, pathological gaming, problematic video gaming, or problematic video game use interchangeably to encompass a continuum from at-risk and problematic to overtly addictive gaming patterns, with the very concept of “addiction” often employed metaphorically or as “addiction-like,” particularly in population-based surveys among youth (5, 23–25).

The search strategy included all available publication types such as original and review articles, conference proceedings, editorials, letters, book chapters, notes, short surveys, retracted articles, and correction, covering the period from 2004, when the first related publication on parenting and IGD was indexed in Scopus and WoS, to 31 December 2024. Such an inclusive strategy was pursued in order to capture the full scope of scholarly communication in this field and is consistent with bibliometric methodologies focused on mapping publication patterns and knowledge development.

Instead of up to the exact search date, we decided to limit the search to a specific date - 31 December 2024 – for several reasons. Primarily because we wanted to limit the search period to exactly 20 years from the emergence of the IGD phenomenon (2004–2024), but also because selecting such an end date ensures a certain time lag, consistency, and prevents the inclusion of partially indexed or recently added records that may not yet be fully processed or categorized within any database. Consequently, the dataset represents a stable, reproducible, and finalized state of indexing as of the end of the 2024 calendar year. Using this time boundary also facilitates the identification of long-term publication patterns and thematic evolution in research on the relationship between parenting and IGD, while enabling comparisons with other bibliometric studies that adopt full-year intervals. Moreover, this aligns with the study’s objective, i.e., to examine recent developments and new trends in recent IGD literature, while ensuring methodological transparency and reproducibility. Finally, it is worth noting that most previous bibliometric analyses in this area relied on a single database (WoS or Scopus), and we are not aware of any that covered multiple ones. Including both databases in this study increases comprehensiveness and robustness of the analysis by minimizing duplication and maximizing coverage across different disciplines.

**Figure 1:**
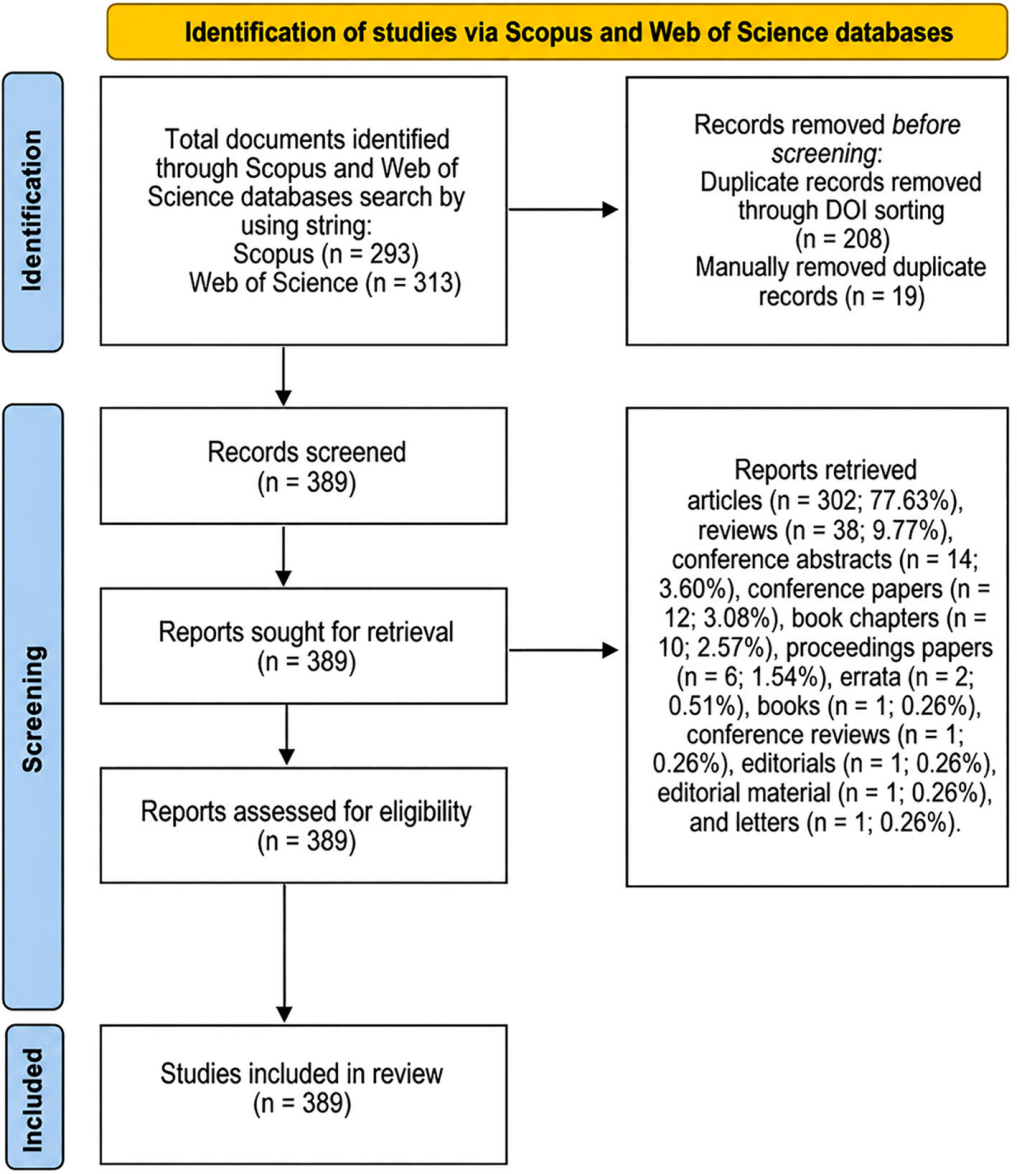
PRISMA flowchart of bibliometric review.

The publication selection process followed the PRISMA 2020 guidelines (26). A PRISMA flow diagram (**Figure 1**) was used to illustrate the phases of identification, screening, eligibility, and inclusion in the bibliometric dataset. There were no other exclusion or inclusion criteria.

### 2.3 Data extraction and analysis

Metadata from the selected publication corpus were extracted and loaded into VOSviewer 1.6.20 (Leiden University, Netherlands) (27), Bibliometrix (5.0) 4.4.2 (28), and Excel (Microsoft Office 365 MSO (Version 2508), Microsoft Corporation, Redmond, WA, USA). As mentioned above, current bibliometric studies in the field of IGD analysis typically retrieved papers exclusively from one database, most often WoS, with one from Scopus.

Recently, Caputo and Kargina presented a transparent and user-friendly method for merging data from Scopus and WoS without any coding or specialized software, using the R package Bibliometrix (29). In this paper, we used the mentioned method because it can be used to conduct a bibliometric study without compromising data integrity - the datasets from Scopus and Web of Science (WoS) were exported in BibTex format and converted to Excel files compatible with Bibliometrix using the biblioshiny function in the RStudio software tool. The two files were then manually merged in Excel by aligning column structures, with one designated as the main dataset, then combined, and the resulting dataset was cleaned through systematic removal of duplicate records (by two separate researchers) and manual checking of the set to prevent redundancy and create a unique dataset for bibliometric analysis. Afterward, we used VOSviewer for bibliometric mapping of keywords defined by the article authors and chronological analysis of the active representation of certain terms. For detailed recognition of data extracted from Scopus and WoS and descriptive analysis related to the number of scientific journals, institutions, countries, and authors, bibliometric techniques supported by the Bibliometrix library functions executed in RStudio were used. In this paper, a descriptive bibliometric analysis, bibliometric mapping, and content analysis were conducted: annual scientific production is presented, the most cited articles were analysed by source, year of publication, article type, country, authorship, etc. The most frequently used authors are also presented and analysed based on their occurrence. Publication sources were ranked by their relevance. For the 10 most cited articles, as well as all articles, the average and median number of citations were examined. Co-authorship and authorship analyses were conducted by calculating the number of multi-author publications versus single-author publications. All analyses were performed using VOSviewer 1.6.20, a software tool specifically developed for this type of research.

## 3 Results

The results of the search using the aforementioned search string yielded a total of 293 papers in Scopus and 323 papers in WoS, totaling 616 papers. All articles were downloaded, extracted, and listed in Excel, and coded based on whether the article was found in WoS (W + serial number) or Scopus (S + serial number). From this dataset, duplicates were identified (n = 208 papers) by sorting articles by DOI identifiers and author names, followed by manual comparison of the existence of two articles with similar details, and exclusion of irrelevant papers from the initial results (n = 19 papers). After the aforementioned exclusions, a total of 389 papers were retrieved and extracted from the Scopus and WoS databases. The types of included papers, sorted by frequency, include: articles (n = 302; 77.63%), reviews (n = 38; 9.77%), conference abstracts (n = 14; 3.60%), conference papers (n = 12; 3.08%), book chapters (n = 10; 2.57%), proceedings papers (n = 6; 1.54%), errata (n = 2; 0.51%), books (n = 1; 0.26%), conference reviews (n = 1; 0.26%), editorials (n = 1; 0.26%), editorial material (n = 1; 0.26%), and letters (n = 1; 0.26%).

### 3.1 Keywords

**Figure 2:**
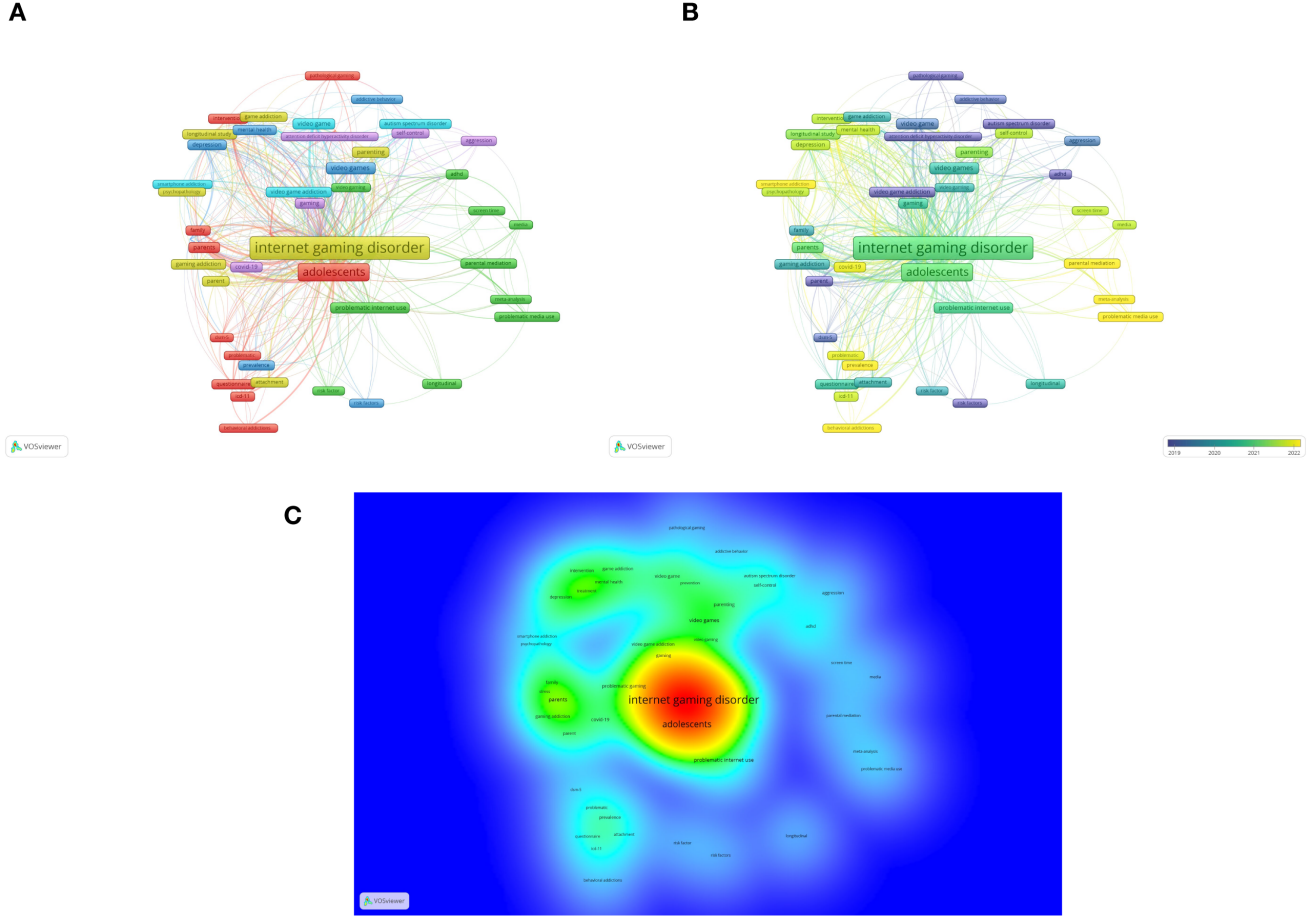
Bibliometric map of research by author keywords **(A)**, trending themes by average publication year **(B)**, density map **(C)**.

A minimum occurrence threshold of 5 was selected for keywords, using the full counting method. Of 904 author keywords, 62 met this criterion. For each of these keywords, the total link strength with other keywords was calculated, and those with the highest total link strength were identified. The ten author keywords with the greatest relevance are: “Internet gaming disorder”, “gaming disorder”, “adolescents”, “Internet addiction”, “addiction”, “adolescent”, “children”, “video game”, “parents”, and “problematic gaming”. Analysis using VOSviewer confirmed the relevance of the search terms and enabled the construction of **Figure 2**, which shows keyword co-occurrence (**Figure 2A**), average publication year per keyword (**Figure 2B**), and density map (**Figure 2C**). Colors in **Figure 2A** indicate clusters formed by keyword co-occurrence, while line thickness shows the number of papers in which they co-occur. Node size represents the number of papers in which the keyword appears; 3 was the minimum occurrence when papers are concerned. Color in **Figure 2B** shows trending themes as the average year of papers in which keywords appear, and color in **Figure 2C** shows paper frequency for each keyword, where larger sizes and more intense colors indicate higher frequency.

### 3.2 Publication year

**Figure 3:**
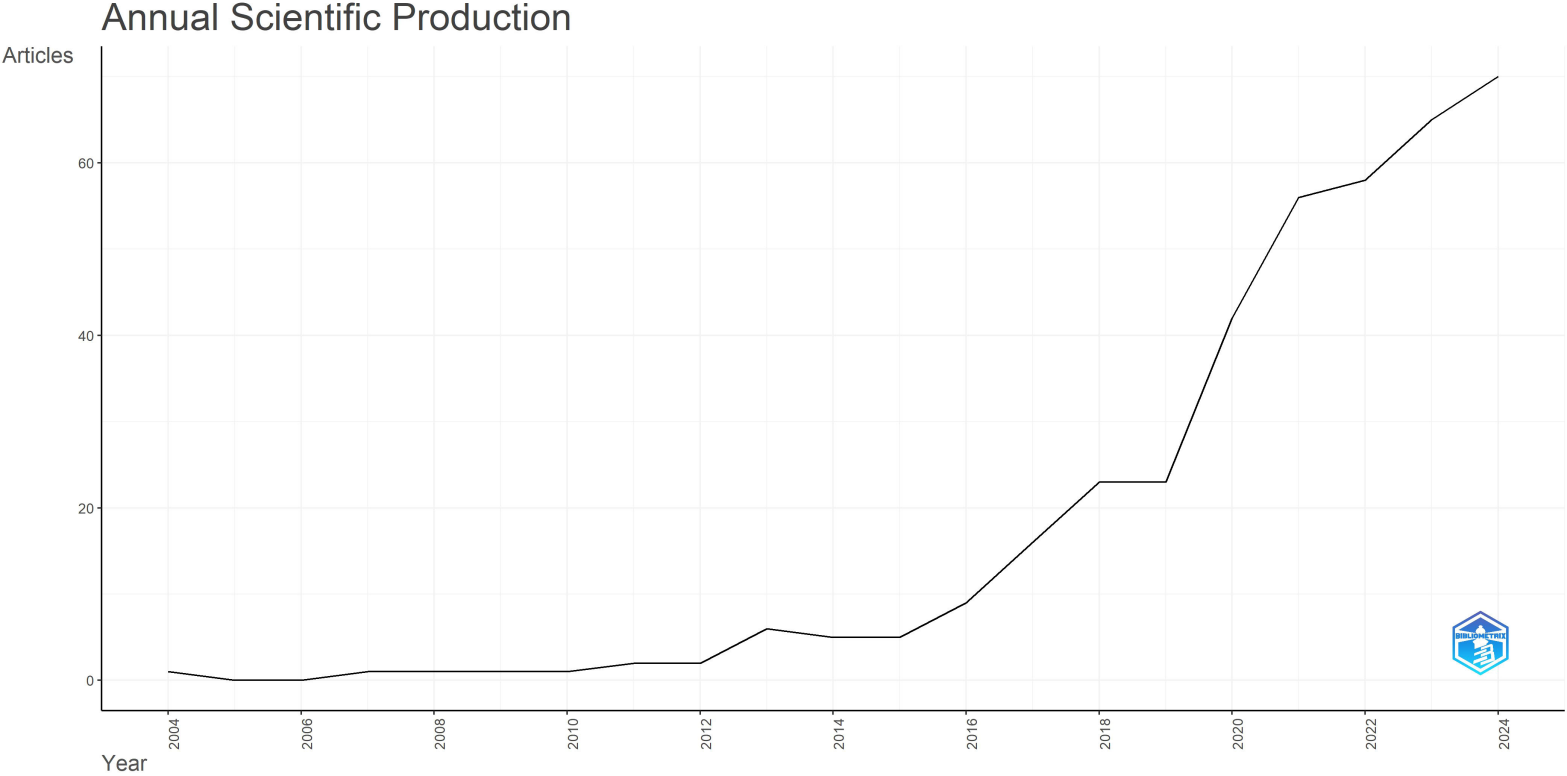
Dynamics of research literature on the relationship between parents and IGD.

The results in **Figure 3** show that scientific interest in the relationship between parenting and IGD began in 2004, with the first paper on this topic, which dealt with research on video game addiction in children and adolescents in Taiwan. Until 2010, typically one study per year was published, and from then on, the topic began to appear more consistently in the literature, with the number of papers growing each year with minor deviations, reaching a peak in the 2024 (n = 70), indicating that the topic remains of interest to researchers. The most significant increase in publications occurred between 2019 and 2020 (from 23 to 42 papers; n = 19).

### 3.3 Display of the 10 most cited articles

**Table 1** shows the 10 most cited articles in the field of the relationship between parents and IGD. With a total of 268 citations to date, the review article titled “Family factors in adolescent problematic Internet gaming: A systematic review” by Luke A. Schneider, published in 2017 in the Journal of Behavioral Addictions, was the most cited article in the mentioned field.

**Table 1:** Top 10 cited literatures.

Rank	First author	Citations	Article title	Journal abbreviation	Date	Article type
1	Schneider, Luke A.	268	Family factors in adolescent problematic Internet gaming: A systematic review	J Behav Addict	2017	Review
2	Chiu, Shao-I	249	Video Game Addiction in Children and Teenagers in Taiwan	Cyberpsychol Behav	2004	Article
3	Mazurek, Micah O.	219	Television, Video Game and Social Media Use Among Children with ASD and Typically Developing Siblings	J Autism Dev Disord	2013	Article
4	Wartberg, Lutz	199	A longitudinal study on psychosocial causes and consequences of Internet gaming disorder in adolescence	Psychol Med	2019	Article
5	Karaer, Yusuf	176	Parenting styles, perceived social support and emotion regulation in adolescents with internet addiction	Compr Psychiat	2019	Article
6	D’Arienzo, Maria Chiara	175	Addiction to Social Media and Attachment Styles: A Systematic Literature Review	Int J Mental Health Addict	2019	Article
7	Király, Orsolya	159	Intense video gaming is not essentially problematic	Psychol Addict Behav	2017	Article
8	Mazurek, Micah O.	154	Video Game Use in Boys With Autism Spectrum Disorder, ADHD, or Typical Development	Pediatrics	2013	Article
9	Domoff, Sarah E.	153	Development and validation of the Problematic Media Use Measure: A parent report measure of screen media “addiction” in children.	Psychol Pop Media Cult	2019	Article
10	Bonnaire, Céline	151	Relationships between parental attitudes, family functioning and Internet gaming disorder in adolescents attending school	Psychiatry Res	2017	Article

Of the 10 most cited papers, one (the aforementioned Schneider paper) is a review, while the other nine are original scientific papers. The 10 most cited articles have an average of 190 citations, with a median of 176. Across all articles, the average number of citations is 24, and the median is 9.

The number of citations ranges from 268 to 151, indicating a certain level of scientific attention/interest and impact within the field. The most cited paper, the systematic review “Family factors in adolescent problematic Internet gaming: A systematic review” by Schneider et al. (2017), emphasizes the central role of family dynamics and parental factors in understanding, preventing, and treating IGD in adolescents (13).

Chronologically, the most cited papers are concentrated between 2013 and 2019, with only one earlier study (Chiu, 2004) marking a pioneering empirical step, i.e., representing the first paper in the field of the relationship between parenting and IGD, which is why it is understandably the second most cited paper. The paper by Chiu et al. (2004) investigates video game addiction among Taiwanese children and adolescents, highlighting its association with hostility, social skills, and academic achievement, while suggesting that current addiction models do not adequately explain these results (30). Mazurek and Engelhardt, in the paper “Television, Video Game and Social Media Use Among Children with ASD and Typically Developing Siblings”, published in 2013 have investigated the association of all screen use in children with neurodevelopmental disorders, and it is one of the first studies on this topic. The results indicate that children with neurodevelopmental disorders use television and video games significantly more and have a higher prevalence of IGD regardless of gender, while social media use results are opposite (31).

The longitudinal study by Wartberg et al.”A longitudinal study on psychosocial causes and consequences of Internet gaming disorder in adolescence” from 2019 is expectedly high on the cited list (199) as it is the first longitudinal study examining risk factors for IGD in adolescents. The most important contribution of the frequently cited paper (176) Parenting styles, perceived social support and emotion regulation in adolescents with internet addiction are results linking lower parental supervision, higher levels of alexithymia, and the presence of anxiety disorders with the development of internet use addiction, suggesting that preventive and therapeutic interventions need to target strengthening the quality of parenting, social support, and emotion regulation in adolescents (32).

D’Arienzo et al. (2019) in a systematic literature review from 2000-2018 “Addiction to Social Media and Attachment Styles: A Systematic Literature Review” pointed to the association between parenting styles and internet addiction. The authors suggest that insecure attachment style is consistently associated with more intense and dysfunctional internet and social media use as a way to compensate for the lack of closeness and support in close relationships (33).

Király et al. in the paper “Intense video gaming is not essentially problematic” empirically investigate the association between gaming intensity and IGD. Their results suggest that gaming time is not necessarily associated with the development of IGD; instead, psychiatric symptoms of the player and motivation, especially escapism, play a more important role (34).

Among the last three papers by citation count, there are no large differences (citation counts 154-151). Mazurek and Engelhardt (2013) in the paper “Video Game Use in Boys With Autism Spectrum Disorder, ADHD, or Typical Development” focus on video game use and IGD in boys with neurodevelopmental disorders (35). Domoff et al. in the paper “Development and validation of the Problematic Media Use Measure: A parent report measure of screen media “addiction” in children” develop and test the Problematic Media Use Measure questionnaire for parents to assess screen addiction in children aged 4–11 years; it is highly reliable and based on DSM-5 criteria for IGD (36).

Bonnaire (2017) conducts research on children’s perception of family relationships and IGD in “Relationships between parental attitudes, family functioning and Internet gaming disorder in adolescents attending school”. Their results indicate that adolescents with IGD experience weaker family connectedness, more conflicts, and poorer family relationships, and that parental attitudes involving reduced parental supervision and lack thereof are risk factors for IGD (37).

This temporal distribution shows that systematic interest in parental correlates of gaming addiction intensified in the past decade, which aligns with the formal recognition of IGD as a clinical condition in DSM-5 (2013) (38).

Thematically, these papers focus on three main areas:

1. Parenting and family environment (37, 39)
2. Comorbid or developmental contexts, particularly autism spectrum disorders and ADHD (31, 35)
3. Broader engagement in digital media, including social networks and general screen exposure/addiction (33, 36)

From a bibliometric perspective, the frequent appearance of journals from the fields of psychology and psychiatry, such as Journal of Behavioral Addictions, Psychological Medicine, and Comprehensive Psychiatry, shows that the relationship between parenting and IGD is primarily framed within behavioural areas of science. The concentration of citations in a relatively narrow temporal and disciplinary framework highlights a mature but still consolidating research field, in which family-based mechanisms are increasingly recognized as protective and risk factors in adolescent video game-related behaviour. Together, the most cited papers form a coherent intellectual foundation linking developmental psychology, family studies, and digital media research, guiding further empirical and preventive studies.

### 3.4 Global distribution of research results and citations

This study included a total of 55 countries. **Table 2** shows the twenty countries contributing most to research on the relationship between parenting and IGD, ranked primarily by total number of articles, supplemented by total and average number of citations and distribution of corresponding authors (CA). The leading countries by research productivity in the mentioned field are China (55 papers), Germany (45), and the United States (29), which together account for a significant share of global production in this area (China 14.1% of total papers, Germany 11.6%, and United States 7.5%) and represent dominant forces in the global scientific collaboration network. Ranking of countries by research output and international collaboration in the top 20 countries based on corresponding authors with full counting was performed by RStudio/Bibliometrix, which identified scientific articles in two groups - SCP and MCP, where SCP includes papers with authors from one country, and MCP includes authors from multiple countries. The primary goal is to understand the scope of global engagement in research, particularly in terms of country collaboration patterns. The balance between SCP and MCP countries represents one of the key bibliometric indicators of national research independence versus international collaboration. The data show that China and Germany lead in total publication volume, reflecting their dominant role in coordinating and producing IGD-related research. Both countries show a strong predominance of SCPs, indicating that most publications are conducted within domestic research networks. This pattern suggests strong internal institutional capacity, consolidated national research programs, and high institutional productivity, but also relatively lower levels of international collaboration.

**Table 2:** Top 20 countries distributed by publications and citations by corresponding author’s countries.

Ranked by publications	Country	Total articles	Citations	Average citations (AC)	Corresponding author (CA)		/389%	Rank by AC
					SCP	MCP		
1	China	55	1298	24	50	5	14,1	=10
2	Germany	45	1092	24	44	1	11,6	=10
3	USA	29	1187	41	21	8	7,5	6
4	South Korea	23	391	17	19	4	5,9	15
5	Hong Kong	17	670	39	8	9	4,4	7
6	France	15	297	20	14	1	3,9	=13
7	Türkiye	15	299	20	15	0	3,9	=13
8	Spain	14	219	16	12	2	3,6	16
9	India	13	53	4	12	1	3,3	20
10	Australia	11	491	45	8	3	2,8	=4
11	Japan	11	286	26	11	0	2,8	9
12	Italy	10	231	23	8	2	2,6	12
13	Sweden	8	115	14	7	1	2,1	17
14	Indonesia	7	64	9	7	0	1,8	19
15	Switzerland	6	272	45	5	1	1,5	=4
16	Canada	5	229	46	4	1	1,3	3
17	Hungary	5	322	64	2	3	1,3	2
18	Israel	4	48	12	3	1	1,0	18
19	Thailand	4	127	32	3	1	1,0	8
20	UK	4	287	72	3	1	1,0	1

In contrast, countries like the United States (27.6% MCP papers), South Korea (17.4% MCP), Hong Kong (52.9% MCP), and Australia (27.3% MCP) show a more balanced ratio between SCP and MCP results. The higher MCP share in these countries reflects stronger participation in international partnerships and cross-cultural studies, which is an important characteristic in a field examining behavioural and family phenomena shaped by cultural and social contexts. Of the top 20 countries by productivity, only Hong Kong and Hungary (60.0%) have more MCP collaboration papers than SCP.

Despite the highest number of papers and citations in China (total 1298 citations) and an average citation count (AC) of 24, it appears their research prioritizes quantity and growing productivity over long-term citation impact. In contrast, the United States, with fewer publications, achieves a higher average citation rate (41) and ranks third in productivity and sixth in average citation rate, indicating comparatively greater global visibility and citation impact per study. The German research community ranks second in number of publications (45 papers, AC = 24), supported by a strong institutional base at universities such as Universitätsklinikum Hamburg-Eppendorf and MSH Medical School Hamburg (as shown in **Table 3**). Germany’s dominance in single-country productivity (n = 44; 97.8%) suggests a strong national research infrastructure with limited reliance on international collaboration (n = 1; 2.2%).

**Table 3:** Top 10 authors distributed by publications.

Rank	Author	Publications	Country	Institution	H-index	PY_start
1	Thomasius, Rainer	16	Germany	Universitätsklinikum Hamburg-Eppendorf	11	2015
2	Paschke, Kerstin	14	Germany	Universitätsklinikum Hamburg-Eppendorf	10	2020
3	Wartberg, Lutz	14	Germany	MSH Medical School Hamburg	9	2015
4	Griffiths, Mark D.	12	United Kingdom	Nottingham Trent University	11	2017
5	Jeong, Hyunsuk	9	South Korea	Catholic University of Korea	7	2017
6	King, Daniel L.	9	Australia	Flinders University	7	2017
7	Lindenberg, Katajun	8	Germany	Goethe-Universität Frankfurt	5	2020
8	Austermann, Maria Isabella	7	Germany	Universitätsklinikum Hamburg-Eppendorf	7	2020
9	Lee, Hae-Kook	7	South Korea	Catholic University of Korea	7	2017
10	Lee, Seungyup	7	South Korea	Catholic University of Korea	6	2017

In contrast, less productive but exceptionally cited research communities such as Switzerland (8 papers; AC = 45), Australia (11 papers; AC = 45), Canada (5 papers; AC = 46), and especially Hungary (5 papers; AC = 64) and the United Kingdom (4 papers; AC = 72) show that countries with fewer publications can still have disproportionate citation impact, often due to methodological innovations or early conceptual contributions. The United Kingdom, ranked twentieth by number of publications but first by average citation rate, represents an example of high-impact/citation papers with low quantity. Meanwhile, South Korea (23 papers; AC = 17) and Hong Kong (17 papers; AC = 39) highlight strong Asian representation and contextual importance of family studies related to gaming/IGD. The SCP/MCP data reveal contrasting collaboration patterns: East Asian and European countries predominantly publish single-country papers, while English-speaking countries (USA, UK, Australia, Canada) show more multinational collaboration and diverse author networks.

**Figure 4:**
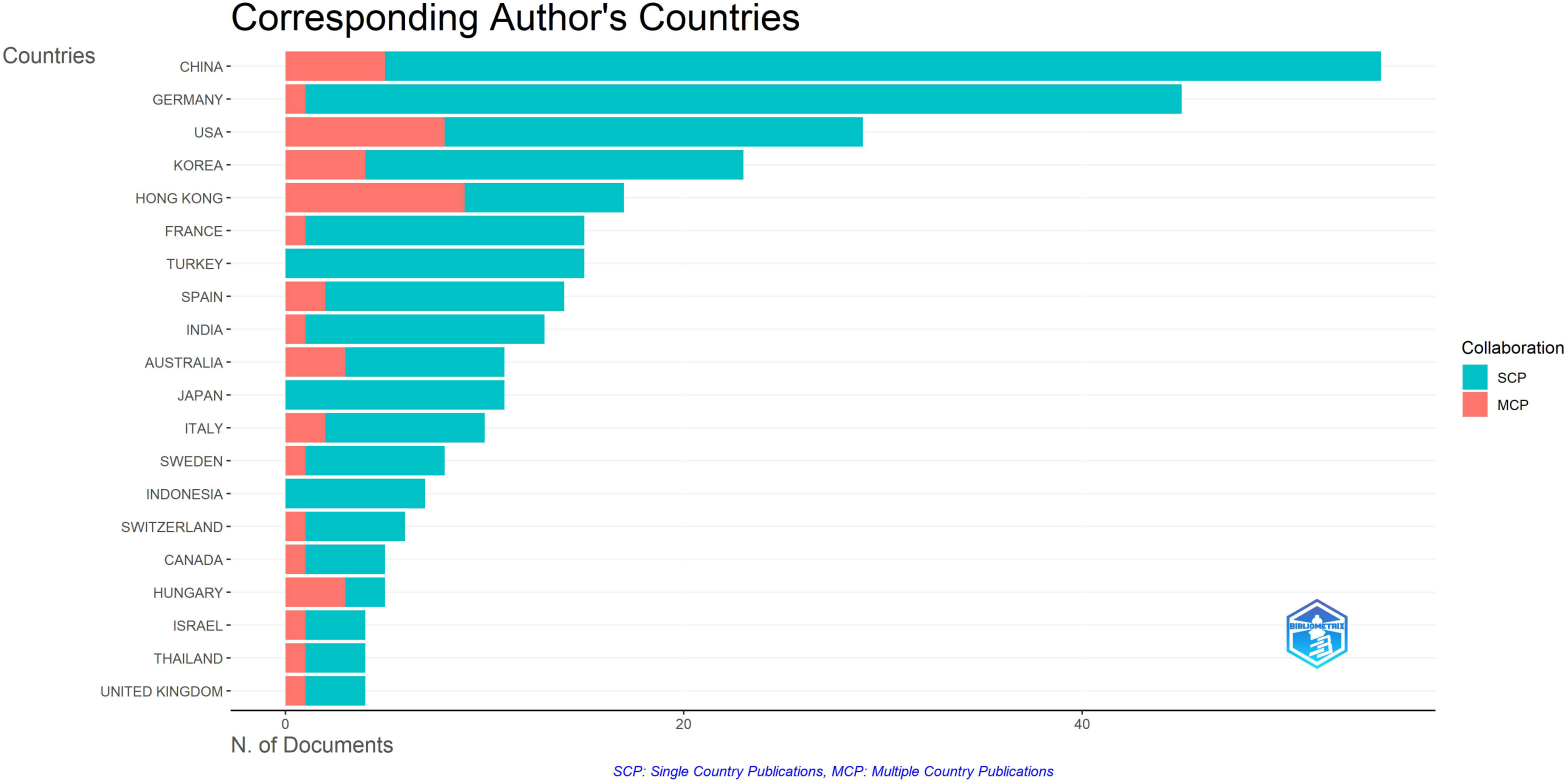
Distribution of publications by single-country publications (SCP) and multi-country publications (MCP).

In summary, **Table 3** illustrates a geographically plural but asymmetric research landscape. East Asian countries (China, South Korea, Hong Kong, Japan) lead in productivity, undoubtedly reflecting regional concern about youth/adolescent gaming behaviour, while Western countries, particularly the United States, United Kingdom, and Canada, show higher citability, emphasizing theoretical and cross-cultural dimensions. The distribution of corresponding authors and average citations together highlights that scientific impact in the field of parenting and interdisciplinary development encompasses international visibility, intensity (inter-country/inter-institutional) collaboration, and theoretical contribution. **Figure 4** shows the distribution of publications according to whether they were published from a single country or through international co-authorship (multiple countries). A clear dominance of Asian countries and Germany is observed, indicating their higher research productivity and/or stronger involvement in international collaborations in the studied field.

**Figure 5:**
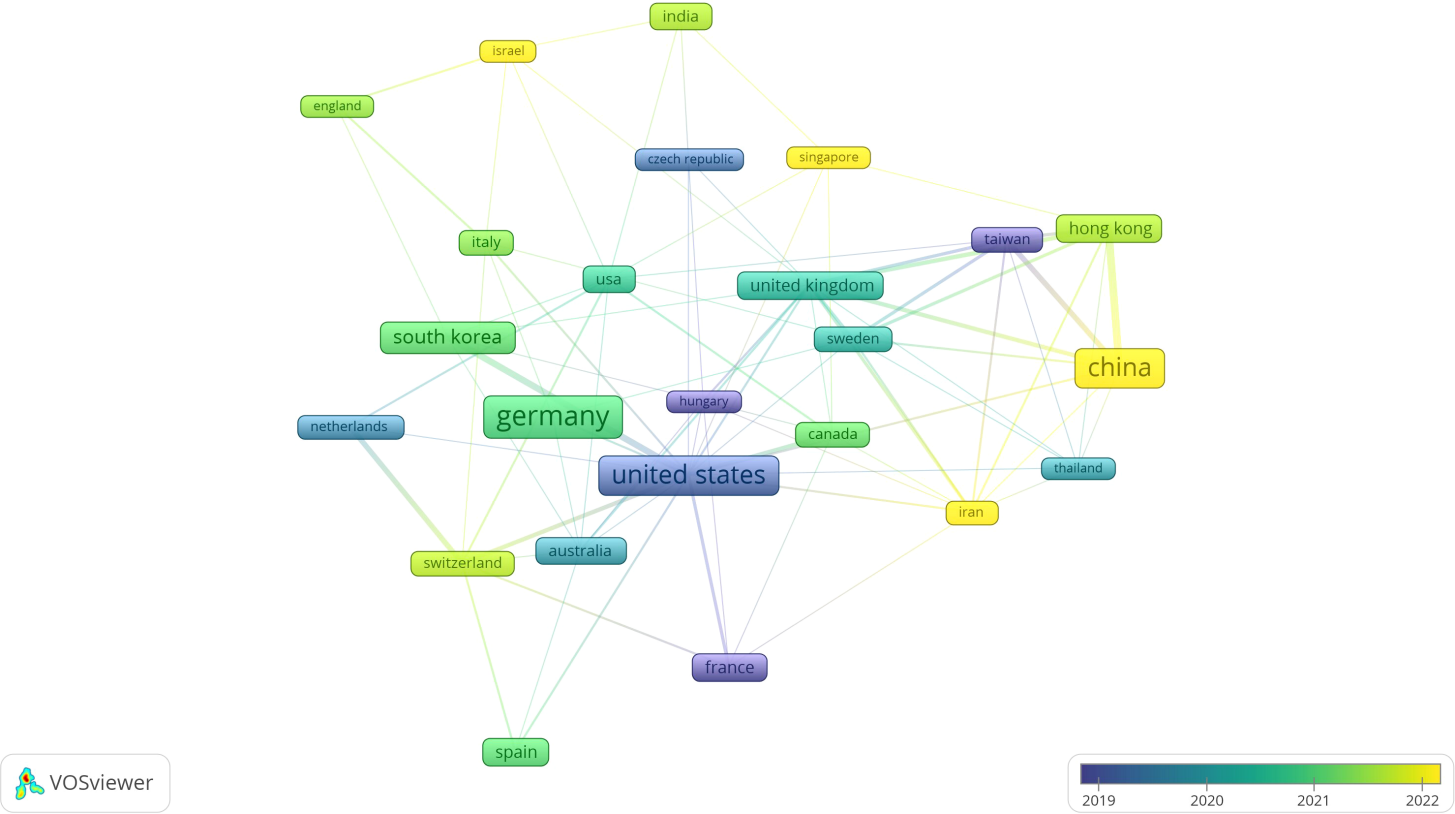
Chronologically structured and labeled collaboration network of the most productive countries.

As can be seen in **Figure 5**, China, Germany, and the USA are the most productive in the field of parenting and IGD papers (frame size represents approximate number of papers) with significant links to other countries, while the legend shows that among these countries, China after 2022 creates the most papers.

**Table 3** shows the ten most productive authors in the field investigating the relationship between parenting and IGD, along with their institutional affiliation, geographic distribution, and academic impact represented by the H-index. The ranking, based on total number of publications, reveals Rainer Thomasius as the leading scientist with 16 papers, followed by Kerstin Paschke and Lutz Wartberg, each with 14 papers. These three authors, all affiliated with German institutions, particularly Universitätsklinikum Hamburg-Eppendorf and MSH Medical School Hamburg, position Hamburg and Germany as a prominent European research hub in the field of parenting and IGD research. Their productivity dating back to 2015 (PY_Start) underscores Germany’s early and continuous leadership in empirical research on family-related determinants of IGD, and it is important to note that among the ten most productive authors in this field, Thomasius and Wartberg have been engaged in this topic the longest. Besides them, two more authors in the top 10 most productive come from Germany, leading to the information that Germany contributes with 50% of the most productive authors. Thus, while the publication count column further highlights the clear dominance of collaborative German teams, it is important to mention Mark D. Griffiths (United Kingdom, Nottingham Trent University), who ranks fourth with 12 papers and represents a central figure in behavioural addiction research, bridging psychological and clinical perspectives; as well as contributions from South Korea and Australia, demonstrating the global relevance of this research topic. Similarly, Daniel L. King from Flinders University in Australia, with nine papers, has significantly contributed to theoretical and diagnostic frameworks of IGD.

The country and institution columns reveal distinct geographic clusters: one concentrated in Germany, characterized by institutional collaboration and clinical-psychological approaches; and another in South Korea, represented by Hyunsuk Jeong, Hae-Kook Lee, and Seungyup Lee (each with seven to nine papers, Catholic University of Korea), which portrays parent-child relationship patterns in IGD cases in East Asia. H-index values range between 5 and 11, suggesting consistent citation impact and scientific visibility, particularly among top-ranked European and Anglo-Australian researchers.

Overall, **Table 3** emphasizes a geographically and quantitatively diverse collaborative network, led by German and South Korean research groups, supplemented by contributions from behavioural scientists from English-speaking regions. The convergence of high productivity and moderate to strong citation indices indicates the maturation of a field grounded in multinational, interdisciplinary efforts linking parenting, psychological changes, and behaviour with digital media/digital content.

Thematic mapping of recent and frequently cited research on parental relationships with IGD reveals six dominant research clusters, each characterized by distinct conceptual foci and methodological orientations. The red cluster encompasses studies addressing the conceptualization and clinical approaches to IGD, emphasizing diagnostic frameworks (DSM-5, ICD-11), intervention and prevention strategies, and operationalization of gaming-related behavioural addictions. Papers with these keywords often highlight the ongoing refinement of IGD as a clinical construct and the role of parental involvement in treatment contexts. The green cluster highlights parental mediation and digital media exposure, focusing on developmental and risk factors in children’s media use. Keywords such as “screen time”, “parental mediation”, and “problematic Internet use” indicate a strong orientation toward environmental determinants of gaming behaviour. The blue cluster focuses on psychological vulnerability and emotional correlates of IGD, situating IGD within broader psychopathological profiles that include depression, anxiety, and stress, often analysed within adolescent and student populations. The yellow cluster reflects an integrative perspective on parenting, attachment, and family psychopathology, emphasizing longitudinal studies and developmental dynamics linking family systems, parenting styles, and addictive behaviours. The violet cluster extends this line of research toward parenting style, self-control, and behaviour regulation, situating IGD in the context of the pandemic (COVID-19), which exacerbated online engagement and IGD. Finally, the teal cluster expands the scope toward digital co-addictions and technology-related behaviours, drawing attention to common mechanisms between, smartphone addiction, and social media, signalling a new interdisciplinary convergence around cross-platform digital addictions. Together, these clusters outline a research landscape bridging diagnostic, psychosocial, and developmental dimensions, emphasizing the central role of parenting as a protective and risk factor in the etiology and maintenance of IGD (**Table 4**).

**Table 4:** Research themes and categories identified in the most recent and highly cited papers, along with their primary research focuses - representative author keywords, categories, and themes in research concerning parental relationship with internet gaming disorder.

Color	Representative author keywords (number of occurences)	Categories	Theme
Red	Adolescents (147), behavioral addictions (9), dsm-5 (22), family (24), gaming disorder (156), icd-11 (16), intervention (15), parents (43), pathological gaming (13), prevention (16), problematic (11), problematic gaming (42), questionnaire (20), treatment (24)	Conceptualization, Diagnosis, Intervention	Conceptualization and Clinical Approaches to IGD
Green	Adhd (21), children (70), Internet (30), longitudinal (8), media (13), meta-analysis (12), parental mediation (12), problematic Internet use (40), problematic media use (12), risk factor (13), screen time (10), video gaming (15)	Parental Mediation, Risk, Developmental Factors	Parental Mediation and Digital Media Exposure & Environment
Blue	Addictive behavior (9), adolescent (90), anxiety (19), depression (26), igd (15), mental health (26), prevalence (23), risk factors (7), stress (22), students (13), video games (38)	Comorbidity, Mental Health Broader Addictive Behaviors	Psychological Vulnerability and Emotional Correlates of IGD
Yellow	Attachment (11), game addiction (12), gaming addiction (23), Internet addiction (92), Internet gaming disorder (181), longitudinal study (19), parent (32), parenting (36), psychopathology (13), youth (14)	Parenting, Addictions, Developmental Dynamics	Parenting, Attachment, and Family Psychopathology
Violet	Adolescence (37), aggression (14), attention deficit hyperactivity disorder (12), covid-19 (33), gaming (31), parenting style (11), self-control (15)	Parenting styles in adolescent behavioral regulation Self-control and aggression, Contextual shifts during the COVID-19 pandemic that intensified gaming behavior.	Parenting Style, Self-Control, and Behavioral Regulation
Teal	Addiction (91), autism spectrum disorder (20), behavioral addiction (25), smartphone addiction (15), social media (20), video game (46), video game addiction (20)	Cross-platform digital addictions Shared behavioral mechanisms with IGD	Digital Co-Addictions and Emerging Technology-Related Behaviors

### 3.5 Journal productivity

**Table 5** shows the ten most productive scientific journals publishing research on the relationship between parenting and IGD, revealing key dissemination channels and citation dynamics within the field. Journal of Behavioral Addictions ranks first with 36 papers, researches on the relationship between parenting and IGD confirming its position as the primary source for behavioural and clinical addiction research. It shows the highest H-index (88) and total citations (1345), with an SJR impact factor of 2.260 and placement in the Q1 quartile in both SJR and JIF (6.2). This combination of productivity, citability, and high quartile ranking emphasizes the journal’s dual role as an essential hub for publication and a citation driver shaping the intellectual core of the parenting and IGD research domain. International Journal of Environmental Research and Public Health follows with 17 papers and 602 citations, reflecting the integration of IGD-related studies into the broader context of public health and family well-being. Despite a relatively modest SJR impact factor (0.919) and Q2 classification, its JIF (4.614, Q1) and high H-index (229) indicate significant academic visibility and multidisciplinary reach. Frontiers in Psychiatry (16 papers) and Frontiers in Psychology (9 papers) represent additional high-impact journals emphasizing clinical, cognitive, and psychosocial aspects of digital media use, with solid H-index values (134 and 212) and consistent placements in Q1-Q2 quartiles across all indexing systems.

**Table 5:** Most prolific journals/top 10 journals distributed by publications and citations.

Journal	Number of publications	H-index	Total citations	Impact factor (SJR - 2024)	Quartile	Journal impact factor (JIF - 2024)
Journal of Behavioral Addictions	36	88	1345	2.260	Q1 (SJR) Q1 (JIF)	6.2
International Journal of Environmental Research and Public Health	17	229	602	0.919	Q2 (SJR) Q1 (JIF)*	4.614*
Frontiers in Psychiatry	16	134	393	1.192	Q1 (SJR) Q2 (JIF)	3.2
Frontiers in Psychology	9	212	247	0.872	Q2 (SJR) Q1 (JIF)	2.9
International Journal of Mental Health and Addiction	8	79	350	1.477	Q1 (SJR) Q2 (JIF)	2.5
Computers in Human Behavior	7	275	94	2.923	Q1 (SJR) Q1 (JIF)	8.9
Addictive Behaviors Reports	6	41	302	1.120	Q1 (SJR) Q2 (JIF)	2.8
Current Psychology	6	69	68	1.024	Q1 (SJR) Q1 (JIF)	2.6
Cyberpsychology, Behavior, and Social Networking	6	190	172	1.330	Q1 (SJR) Q1 (JIF)	3.9
Addictive Behaviors	5	158	154	1.638	Q1 (SJR) Q1 (JIF)	3.6

*Journal pending in WoS in June 2025; data from 2021.

Average number of papers for the 10 most cited journals is 12, with a median of 8. Average H-index for the 20 most cited journals is 148, with a median of 146. Average SJR impact factor for the 10 most cited journals is 1.480, with a median of 1.261. Most cited journal is Journal of Behavioral Addictions with a total of 1345 citations. Average JIF for the 10 most cited journals is 4.12, with a median of 3.40. SJR and H-index data were obtained from SCImago, while impact factor (IF) values and quartile rankings were taken from Journal Citation Reports (JCR), both available as of the study date. Finally, it is important to note that the papers included in this study covered a total of 1389 authors.

**Figure 6:**
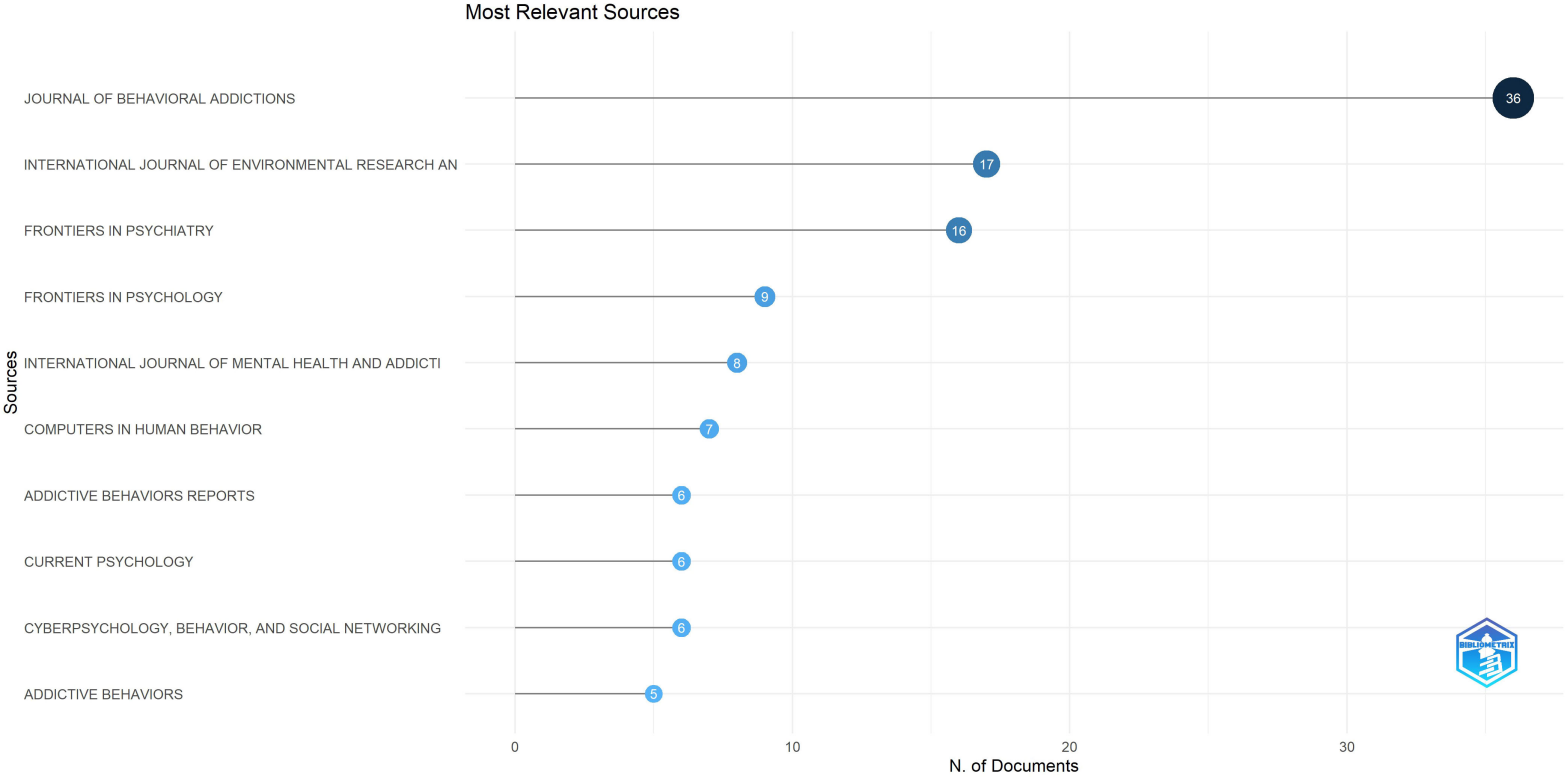
Top 10 most productive journals in the field of parenting and IGD.

The fact that almost all journals in **Table 5** are ranked in Q1 according to SCImago Journal Rank (SJR) or Journal Impact Factor (JIF) indicates that research on parental and family correlates of IGD is published in highly influential, top-tier scientific outlets. Q1 journals represent the top 25% of journals within their subject areas, reflecting high visibility, rigorous peer-review standards, and high citability. This distribution suggests that studies at the intersection of parenting and IGD are recognized as scientifically significant, legitimate, and methodologically robust, attracting publication in leading international journals focused on psychology, psychiatry, and behavioural sciences (**Figure 6**).

## 4 Discussion

The results of the bibliometric analysis indicate that the issue of the relationship between parenting and IGD has evolved over the past two decades into an interdisciplinary topic that continues to develop. This development was driven by the recognition of IGD as a mental disorder, as evidenced by the significant growth in research production from 2004 to 2024, with 389 publications indexed in Scopus and Web of Science databases. The large number of publications, including 302 original articles (77.52%) and 38 reviews (9.82%), highlights interest in family factors of IGD and underscores the interdisciplinarity of the problem. Indeed, among the 10 most cited papers (average 190 citations, median 176), the majority come from *Journal of Behavioral Addictions* (J Behav Addict; Schneider 2017, 268 citations), *Comprehensive Psychiatry* (Compr Psychiat; Karaer 2019, 176), *Psychiatry Research* (Bonnaire 2017, 151), and *Psychological Medicine* (Psychol Med; Wartberg 2019, 199), emphasizing the breadth of the topic and the need to approach the problem from various social and biomedical science fields, particularly psychology, psychiatry, and public health.

Publication trends show slow growth until 2010 (1–2 papers annually), acceleration after IGD inclusion in DSM-5, 2013 (38), peaking in 2024 (70 papers), and a particularly sharp increase from 2019-2020 (23 to 42 papers). The average number of citations is 24 (median 9), indicating growing impact, which was, among other things, driven by the COVID-19 pandemic and rising concern about children’s and adolescents’ exposure to virtual content. In addition to the formal recognition of IGD as a behavioural disorder, this growth is comparable to the increasing prevalence of gaming, particularly in Asia, where video gaming is highly popular. In some Asian countries, video gaming is encouraged and often seen as a source of national pride, with competition wins richly rewarded (40).

The observed increase in publications after 2013, when IGD was recognized as an entity of interest in DSM-5, and especially after 2019 when it was recognized as a mental disorder in ICD-11, can also be explained by improvements in research methodology through the introduction of standardized methodological tools that enable evidence-based knowledge and comparability of results in studies worldwide, given that gaming, like IGD, is not limited to one part of the world but has global interest (41).

The geographic distribution of published research shows dominance of China (55 papers), Germany (45), and the USA (29), with German scientists Rainer Thomasius (16 papers), Lutz Wartberg (14), and Kerstin Paschke (14) dominating among authors. High production in the East Asian region likely reflects the high social relevance of the problem and institutional support for video gaming research, while higher average citability of Anglosphere countries may indicate greater international visibility, stronger theoretical contribution, or publications in journals with broader reach. However, bibliometric citation indicators cannot be interpreted solely as direct indicators of quality, as citability also depends on publication language, open access, co-authorship networks, and disciplinary citation preferences.

In this sense, additional insights into the relationship between SCP and MCP publications deepen understanding of result distribution. The predominance of SCP papers in China and Germany can be interpreted as an indicator of strong national research infrastructure. In contrast, a higher MCP share in the USA, Hong Kong, and Australia may reflect stronger engagement in international partnerships and cross-cultural research, which is particularly relevant for a field studying phenomena sensitive to cultural norms of parenting, family structures, and gaming habits. Additionally, the fact that certain countries with fewer papers record exceptionally high average citability may indicate the presence of several high-impact publications, such as methodologically innovative papers, theoretical syntheses, or papers with high generalizability, which disproportionately shape the field’s citation structure. These results confirm and extend findings from previous bibliometric reviews on IGD (14, 15), but here, parenting is highlighted for the first time as a central thematic cluster, with keywords like “parents”, “parenting styles”, and “parental mediation” dominating in five identified clusters: IGD conceptualization, mediation, mental health, attachment, and digital co-addictions. The most cited papers, led by Schneider’s systematic review (2017, 268 citations), empirically confirm that dysfunctional family relationships characterized by weak cohesion and low expressed warmth predict higher IGD risk, supporting integrative models like Parental Acceptance-Rejection Theory (42) and Family Systems Theory (43). Keyword thematic analysis reveals a shift in research focus from DSM-5 and ICD-11 diagnostic criteria toward preventive interventions, but also the importance of situational themes like COVID-19 impact (44) and psychiatric comorbidities (45) and neurodevelopmental disorders (35). The fact that the most cited paper is a systematic review on family factors (Schneider et al., 2017) (13) suggests recognition of the importance of family relationships in IGD prevention and treatment and that the topic is attractive to a large number of researchers, resulting in a significant scope of results requiring synthesis. Furthermore, the high citability of the first published paper (30) indicates that early attempts at conceptualizing video game addiction focused on outcomes like hostility, social skills, or academic achievement and questioned original addiction models, opening space for later development of specific IGD criteria. This shift from general internet addiction criteria to specific IGD criteria is important as it directs discussion toward the need for an interdisciplinary approach in prevention, including parents, schools, and the community.

Among the most cited papers, the longitudinal study by Wartberg et al. (2019) stands out (32). Longitudinal designs play a key role in the IGD field as they enable disentangling temporal sequences and highlight the question of whether family conflicts and lower warmth predict increased expression of criteria, or vice versa, the presence of criteria and meeting conditions for IGD leads to deterioration of family communication and increased conflicts.

These results indicate that parenting is a important theme in the IGD literature, but it should be interpreted within a broader socio-ecological framework. Research shows that risk and resilience for IGD are shaped at the individual, family, peer, school/institutional, and socio-political levels, implying that the effects of parental monitoring, warmth, and cohesion depend on the context in which they are enacted and that prevention should not remain limited to the family. For this reason, IGD prevention and treatment should involve public health and legislative policies, schools, and the local community, because risky gaming patterns are shaped and maintained through institutional rules, the availability of support, school-related stressors, as well as peer and community norms (46).Previous analyses, such as Zhang & Park (2023) (WoS, 2009-2021; focus: trends, keywords, countries), identify China and the USA as leaders but without emphasis on parental factors (which appear only peripherally) (16). Similarly, Li et al. (2023) (Scopus/WoS; clusters: psychopathology, neuroscience) do not address family dynamics in depth, while Hanoum et al. (2025) (21) uses only Scopus for gaming addiction (528 papers, 2014-2025). Our work surpasses the mentioned analyses by using two databases (389 papers from Scopus and Web of Science) and thematic depth encompassing five clusters with parenting at the centre. However, our study also has several limitations. Using two databases, Scopus and WoS, increases coverage and reduces single-database bias but may overlook papers published outside these two databases (47).

A key interpretive question these findings raise is how to link the bibliometric “picture of the field” with clinical and public health practice. Dominance of themes like prevention, treatment, parental mediation, and mental health suggests research is increasingly seeking applicable intervention models treating parents as active partners through parenting programs, communication training, structuring free time, and digital habits. Therefore, the discussion should emphasize that preventive measures must go beyond “screen time” limits to include developing self-regulation, emotional literacy, and family cohesion.

Identified gaps in the literature include the need for longitudinal studies examining the relationship between family context, motivation, and personal character traits with IGD, with particular emphasis on causal relationships. There is also a need for research in countries where family relationships and IGD topics are less explored, such as Croatia and neighboring countries, as cultural and socioeconomic specifics may influence gaming patterns, parenting practices, and intervention availability.

The findings of this bibliometric analysis should be interpreted within a body of literature primarily focused on “parenting” (e.g., parenting styles, parental mediation, monitoring, and warmth), because such a focus inevitably shapes the “picture” of the research field reconstructed by bibliometric methods. Although this approach is justified by the study aim, it also constitutes a limitation. The selected keywords and search strategy likely did not fully capture studies that describe relevant family influences using different terminology. As a result, some complementary perspectives may be underrepresented, particularly those viewing IGD through the lens of family systems, social support, or a broader family context rather than directly through parenting constructs. We therefore recommend that future bibliometric studies broaden their search by incorporating wider family-related terminology (e.g., family environment, family functioning, family dynamics, caregivers) to capture diverse theoretical traditions and ensure a more comprehensive integration of evidence on family factors in IGD research.

Aggregating results from studies that operationalize IGD using partly different diagnostic frameworks - DSM-5/DSM-5-TR, which is more symptom-oriented (5), and ICD-11, which is oriented toward functional impairment (3) - can systematically obscure subtle but conceptually important differences in research. When such heterogeneous criteria are treated as equivalent, analyses may inadvertently merge populations with different levels of clinical severity and increase result heterogeneity, which may represent a limitation of this study. It is methodologically justified to suggest that future bibliometric studies conduct stratified analyses by diagnostic framework.

Future bibliometric studies in the field of IGD and parental factors should include additional professional/biomedical databases like PubMed and MEDLINE to improve thematic coverage. Additionally, expanding search strategies to include broader terminology related to parents/family (e.g., family environment, caregiving practices, parental mediation, etc.) would further enhance the comprehensiveness of retrieved records. It also has to be noted that citation-based indicators may be influenced by factors such as journal visibility and open-access status, rather than reflecting only the scientific quality and/or impact of individual studies.

Moreover, future studies could benefit from conducting comparative bibliometric analyses in different geographic regions; this is not only due to potential language bias that may lead to underrepresentation of research conducted in non-English-speaking context, but also because it would enable examination of parenting and IGD in specific socio-cultural contexts conditioned by differences in lifestyle and behaviors, socioeconomic factors, and health practices – thereby providing deeper insights into cultural differences in researching these phenomena.

Finally, combining bibliometric mapping with narrative reviews of the most influential papers and publications could bridge quantitative trends with qualitative interpretation, strengthening the relevance of bibliometric findings. By mapping the development and main themes of this research field, this study provides a clear foundation for future longitudinal research and the development of family-based prevention and intervention strategies for IGD.

## 5 Conclusion

The results of this bibliometric analysis show that research on the relationship between parenting and IGD has experienced clear quantitative and thematic growth over the past two decades, with particular intensification following its formal inclusion in DSM-5 and ICD-11. A steady increase in publications was observed, concentrated in psychiatric and psychological journals, with prominent research centres in China, Germany, the USA, and East Asian countries taking a leading role in global production. The results suggest that lower parental warmth, dysfunctional family relationships, and lack of parental control, combined with psychiatric comorbidities and emotional dysregulation, are associated with higher IGD risk, while warmer, supervising, and supportive family relationships can be linked to lower risk. Thematic mapping reveals several stable research clusters: from IGD conceptualization and diagnosis, through parental mediation and digital media exposure, to comorbidities, attachment, and other technology-related behavioural disorders - confirming that IGD is increasingly seen as the result of interactions among numerous individual, family, and social factors. Despite significant progress in developing this research field, gaps remain, with the notable lack of longitudinal studies.

## Data Availability

The original contributions presented in the study are included in the article/supplementary material. Further inquiries can be directed to the corresponding author.

## References

[1] Ko Y-M Lee ES Park S . Prevalence, correlates, and comorbidities of internet gaming disorder and problematic game use: national mental health survey of Korea 2021. Front Psychiatry. (2024) 15:1442224. doi: 10.3389/fpsyt.2024.1442224. PMID: 39473915 PMC11518804

[2] Satapathy P Khatib MN Balaraman AK R R Kaur M Srivastava M . Burden of gaming disorder among adolescents: A systemic review and meta-analysis. Public Health Pract. (2025) 9:100565. doi: 10.1016/j.puhip.2024.100565. PMID: 40115446 PMC11925544

[3] Organization WH . ICD-11: international classification of diseases 11th revision: the global standard for diagnostic health information. Available online at: https://books.google.hr/books?id=H8WFzgEACAAJ (Accessed March 15, 2026).

[4] APA . Diagnostic and statistical manual of mental disorders: DSM-5 2013. Geneva: American Psychiatric Publishing (2013).

[5] American Psychiatric Association . DSM-5-TR. In: Diagnostic and statistical manual of mental disorders. Arlington, VA: American Psychiatric Association Publishing (2022). doi: 10.1176/appi.books.9780890425787

[6] Stevens MWR Delfabbro PH King DL . Prevention strategies to address problematic gaming: An evaluation of strategy support among habitual and problem gamers. J Prim Prev. (2021) 42:183–201. doi: 10.1007/s10935-021-00629-0. PMID: 33710442 PMC7970787

[7] Zhou J Zhao H Wang L Zhu D . The vicious cycle of family dysfunction and problematic gaming and the mediating role of self-concept clarity among early adolescents: A within-person analysis using random intercept cross-lagged panel modeling. J Behav Addict. (2023) 12:920–37. doi: 10.1556/2006.2023.00054. PMID: 38141062 PMC10786229

[8] Fam JY . Prevalence of internet gaming disorder in adolescents: A meta‐analysis across three decades. Scand J Psychol. (2018) 59:524–31. doi: 10.1111/sjop.12459. PMID: 30004118

[9] Gou S Zhang W Tang Y Zhang J He Q . Prevalence of internet gaming disorder among Chinese adolescents: A systematic review and meta-analysis. Asian J Psychiatry. (2024) 102:104257. doi: 10.1016/j.ajp.2024.104257. PMID: 39366164

[10] Rho M Lee H Lee T-H Cho H Jung D Kim D-J . Risk factors for internet gaming disorder: Psychological factors and internet gaming characteristics. Int J Environ Res Public Health. (2017) 15:40. doi: 10.3390/ijerph15010040. PMID: 29280953 PMC5800139

[11] Wang Y Liu B Zhang L Zhang P . Anxiety, depression, and stress are associated with internet gaming disorder during COVID-19: Fear of missing out as a mediator. Front Psychiatry. (2022) 13:827519. doi: 10.3389/fpsyt.2022.827519. PMID: 35222126 PMC8873089

[12] Tsui YY Cheng C . Internet gaming disorder, risky online behaviour, and mental health in Hong Kong adolescents: The beneficial role of psychological resilience. Front Psychiatry. (2021) 12:722353. doi: 10.3389/fpsyt.2021.722353. PMID: 34721101 PMC8554051

[13] Schneider L King D Delfabbro P . Family factors in adolescent problematic internet gaming: A systematic review. J Behav Addict. (2017) 6:1–13. doi: 10.1556/2006.6.2017.035. PMID: 28762279 PMC5700711

[14] Qi-Yu Sun S Chiu-Yan Tang A Wang Q Yuet-Foon Chung L Lai-Tong Lee R . A scientometric analysis and critical review of internet gaming disorder behaviours. Int J Ment Health Promot. (2022) 24:795–810. doi: 10.32604/ijmhp.2022.024841

[15] Li T Tang Y . Visualizing the evolution of gaming disorder research: A global literature mining analysis. Front Educ Psychol. (2023) 4. doi: 10.38007/JEP.2023.040108

[16] Zhang MWB Park SY . A bibliometric analysis of research into internet gaming disorders in Korea. Int J Environ Res Public Health. (2023) 20:3786. doi: 10.3390/ijerph20053786. PMID: 36900797 PMC10001575

[17] Oraon AD Saikia S Verma MK . Exploring emerging trends and performance metrics in internet gaming disorder (IGD): a systematic review based on computational mapping. Glob Knowl Mem Commun. (2025). doi: 10.1108/GKMC-06-2024-0377. PMID: 35579975

[18] Cheng X Fan Y Li S Li X Jin S Zhou C . Research landscape and trends of internet addiction disorder: A comprehensive bibliometric analysis of publications in the past 20 years. Digit Health. (2025) 11:20552076251336940. doi: 10.1177/20552076251336940. PMID: 40297375 PMC12034966

[19] Muflih S Al-Azzam SI Alzoubi KH Karasneh R Hawamdeh S Sweileh WM . A bibliometric analysis of global trends in internet addiction publications from 1996 to 2022. Inform Med Unlocked. (2024) 47:101484. doi: 10.1016/j.imu.2024.101484. PMID: 38826717

[20] Le B-H Nguyen M-Q Binh LTH Hoang A-D . A bibliometrics review of research on internet use disorders since 1996 to 2024. (2024). doi: 10.31219/osf.io/pvuqn

[21] Hanoum M M Hikmah N Alsa A Rahayu A . Mapping the landscape of internet gaming disorder: A comprehensive bibliometric analysis. TPM Test Psychom Methodol Appl Psychol. (2025) 32(S2):12201229. doi: 10.5281/Zenodo.17454487

[22] Liu W . The data source of this study is Web of Science Core Collection? Not enough. Scientometrics. (2019) 121:1815–24. doi: 10.1007/s11192-019-03238-1. PMID: 30311153

[23] Pontes HM Stavropoulos V Griffiths MD . Emerging insights on internet gaming disorder: Conceptual and measurement issues. Addict Behav Rep. (2020) 11:100242. doi: 10.1016/j.abrep.2019.100242. PMID: 32467831 PMC7244902

[24] André F Broman N Håkansson A Claesdotter-Knutsson E . Gaming addiction, problematic gaming and engaged gaming – Prevalence and associated characteristics. Addict Behav Rep. (2020) 12:100324. doi: 10.1016/j.abrep.2020.100324. PMID: 33354616 PMC7744933

[25] Musetti A Floros G Chiappedi M Stavropoulos V . Gaming disorder in the ICD-11: the state of the game. BMC Psychiatry. (2025) 25:1114. doi: 10.1186/s12888-025-07576-8. PMID: 41272537 PMC12640003

[26] Page MJ McKenzie JE Bossuyt PM Boutron I Hoffmann TC Mulrow CD . The PRISMA 2020 statement: an updated guideline for reporting systematic reviews. BMJ. (2021), n71. doi: 10.1136/bmj.n71. PMID: 33782057 PMC8005924

[27] Van Eck NJ Waltman L . VOSviewer manual (2023). Available online at: https://www.vosviewer.com/documentation/Manual_VOSviewer_1.6.19.pdf (Accessed March 15, 2026).

[28] Aria M Cuccurullo C . bibliometrix: An R-tool for comprehensive science mapping analysis. J Informetr. (2017) 11:959–75. doi: 10.1016/j.joi.2017.08.007. PMID: 38826717

[29] Caputo A Kargina M . A user-friendly method to merge Scopus and Web of Science data during bibliometric analysis. J Mark Anal. (2022) 10:82–8. doi: 10.1057/s41270-021-00142-7

[30] Chiu S-I Lee J-Z Huang D-H . Video game addiction in children and teenagers in Taiwan. Cyberpsychol Behav. (2004) 7:571–81. doi: 10.1089/cpb.2004.7.571. PMID: 15667052

[31] Mazurek MO Wenstrup C . Television, video game and social media use among children with ASD and typically developing siblings. J Autism Dev Disord. (2013) 43:1258–71. doi: 10.1007/s10803-012-1659-9. PMID: 23001767

[32] Wartberg L Kriston L Zieglmeier M Lincoln T Kammerl R . A longitudinal study on psychosocial causes and consequences of internet gaming disorder in adolescence. Psychol Med. (2019) 49:287–94. doi: 10.1017/S003329171800082X. PMID: 29622057

[33] D’Arienzo MC Boursier V Griffiths MD . Addiction to social media and attachment styles: A systematic literature review. Int J Ment Health Addict. (2019) 17:1094–118. doi: 10.1007/s11469-019-00082-5. PMID: 30311153

[34] Király O Tóth D Urbán R Demetrovics Z Maraz A . Intense video gaming is not essentially problematic. Psychol Addict Behav. (2017) 31:807–17. doi: 10.1037/adb0000316. PMID: 28956935

[35] Mazurek MO Engelhardt CR . Video game use in boys with autism spectrum disorder, ADHD, or typical development. Pediatrics. (2013) 132:260–6. doi: 10.1542/peds.2012-3956. PMID: 23897915

[36] Domoff SE Harrison K Gearhardt AN Gentile DA Lumeng JC Miller AL . Development and validation of the Problematic Media Use Measure: A parent report measure of screen media “addiction” in children. Psychol Pop Media Cult. (2019) 8:2–11. doi: 10.1037/ppm0000163. PMID: 30873299 PMC6411079

[37] Bonnaire C Phan O . Relationships between parental attitudes, family functioning and internet gaming disorder in adolescents attending school. Psychiatry Res. (2017) 255:104–10. doi: 10.1016/j.psychres.2017.05.030. PMID: 28535475

[38] Borges G Orozco R Benjet C Martínez KIM Contreras EV Pérez ALJ . (Internet) gaming disorder in DSM-5 and ICD-11: A case of the glass half empty or half full: (Internet) Le trouble du jeu dans le DSM-5 et la CIM-11: Un cas de verre à moitié vide et à moitié plein. Can J Psychiatry. (2021) 66:477–84. doi: 10.1177/0706743720948431. PMID: 32806957 PMC8107956

[39] Karaer Y Akdemir D . Parenting styles, perceived social support and emotion regulation in adolescents with internet addiction. Compr Psychiatry. (2019) 92:22–7. doi: 10.1016/j.comppsych.2019.03.003. PMID: 31003724

[40] Anh PQ . Shifting the focus to East and Southeast Asia: A critical review of regional game research. Fudan J Humanit Soc Sci. (2021) 14:173–96. doi: 10.1007/s40647-021-00317-7. PMID: 30311153

[41] Long J Bhad R Potenza MN Orsolini L Phan V Kanabar M . Public health approaches and policy changes after the inclusion of gaming disorder in ICD-11: global needs. BJPsych Int. (2022) 19:63–6. doi: 10.1192/bji.2021.57. PMID: 36287819 PMC9540648

[42] Rohner RP . Introduction to interpersonal acceptance-rejection theory (IPARTheory) and evidence. Online Read Psychol Cult. (2021) 6:3–25. doi: 10.9707/2307-0919.1055

[43] Brown J Errington L . Bowen family systems theory and practice: Illustration and critique revisited. Aust N Z J Fam Ther. (2024) 45:135–55. doi: 10.1002/anzf.1589. PMID: 41531421

[44] Gopali L Dhital R Koirala R Shrestha T Bhusal S Rimal R . Effect of COVID-19 pandemic on internet gaming disorder among general population: A systematic review and meta-analysis. PloS Glob Public Health. (2023) 3:e0001783. doi: 10.1371/journal.pgph.0001783. PMID: 37027365 PMC10081738

[45] Coutelle R Balzer J Rolling J Lalanne L . Problematic gaming, psychiatric comorbidities, and adolescence: A systematic review of the literature. Addict Behav. (2024) 157:108091. doi: 10.1016/j.addbeh.2024.108091. PMID: 38901145

[46] Geudens M De Cock R Zaman B Dupont B . Rethinking gaming disorder prevention: A socio-ecological model based on practitioner insights. Int J Environ Res Public Health. (2026) 23:117. doi: 10.3390/ijerph23010117. PMID: 41595911 PMC12841167

[47] Wilder EI Walters WH . Using conventional bibliographic databases for social science research: Web of Science and Scopus are not the only options. Sch Assess Rep. (2021) 3:4. doi: 10.29024/sar.36

